# [(2*R*,5*R*,6*S*,9*R*)-6-Isopropyl-9-methyl-1,4-dioxaspiro­[4.5]decan-2-yl]methyl 4-bromo­benzoate

**DOI:** 10.1107/S1600536811006428

**Published:** 2011-02-26

**Authors:** Anthony Kiessling, Matthias Zeller

**Affiliations:** a Department of Chemistry and Physics, Mansfield University, Mansfield, PA 16933, USA; b Youngstown State University, One University Plaza, Youngstown, Ohio 44555-3663, USA

## Abstract

The title compound, C_20_H_27_BrO_4_, a 4-bromo­benzoyl derivative of a stereoisomer of glycerol menthonide, synthesized as part of a study of 3-carbon stereochemical moieties, crystallizes with two crystallographically independent mol­ecules in the asymmetric unit, the two mol­ecules differing only in one of the C—O—C—C torsion angles around the ester O atom [−106.5 (7) and 146.1 (6)°]. The two mol­ecules are crystallographically related by a pseudotranslation along the (011) diagonal of the unit cell, emulating a primitive monoclinic cell of half the volume. The translational symmetry is broken by the 4-bromo­benzoate groups. The crystallographic assignment of the absolute stereochemistry is consistent with having started with (−)-menthone, the acetal C atom is *R* and the secondary alcohol is *R*. This brings the bromo­benzoate into approximately the same plane as the menthyl ring and *cis* to the isopropyl group. The glycerol menthonide sections of the molecules interact with each other *via* C—H⋯O interactions, leading to the formation of chains either *A* or *B* molecules that stretch parallel to [010], forming column-shaped double chains. Interactions between neighboring columns are limited to van der Waals contacts.

## Related literature

For the original synthesis of glycerol menthonides, see: Greenberg (1999 ▶). For general background to glycerol menthonides, see: Kiessling *et al.* (2009*b* ▶). For a related structure, see: Kiessling *et al.* (2009*a* ▶).
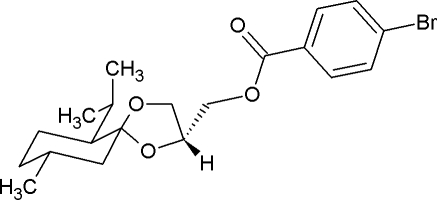


## Experimental

### 

#### Crystal data

C_20_H_27_BrO_4_
*M* *_r_* = 411.33
Monoclinic, 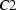

*a* = 42.976 (7) Å
*b* = 5.5763 (9) Å
*c* = 16.072 (3) Å
β = 92.618 (2)°
*V* = 3847.5 (11) Å^3^
*Z* = 8
Mo *K*α radiation
μ = 2.16 mm^−1^
*T* = 100 K
0.50 × 0.05 × 0.03 mm

#### Data collection

Bruker SMART APEX CCD diffractometer
Absorption correction: multi-scan (*SADABS*; Bruker, 2009 ▶) *T*_min_ = 0.588, *T*_max_ = 0.746
17537 measured reflections
9230 independent reflections
6765 reflections with *I* > 2σ(*I*)
*R*_int_ = 0.043

#### Refinement

*R*[*F* ^2^ > 2σ(*F* ^2^)] = 0.058
*wR*(*F* ^2^) = 0.148
*S* = 1.02
9230 reflections
457 parameters
1 restraint
H-atom parameters constrained
Δρ_max_ = 4.00 e Å^−3^
Δρ_min_ = −0.75 e Å^−3^
Absolute structure: Flack (1983 ▶), 3965 Friedel pairs
Flack parameter: 0.000 (13)

### 

Data collection: *APEX2* (Bruker, 2009 ▶); cell refinement: *SAINT* (Bruker, 2009 ▶); data reduction: *SAINT*; program(s) used to solve structure: *SHELXTL* (Sheldrick, 2008 ▶); program(s) used to refine structure: *SHELXTL*; molecular graphics: *Mercury* (Macrae *et al.*, 2008 ▶); software used to prepare material for publication: *SHELXTL* and *Mercury*.

## Supplementary Material

Crystal structure: contains datablocks I, global. DOI: 10.1107/S1600536811006428/tk2718sup1.cif

Structure factors: contains datablocks I. DOI: 10.1107/S1600536811006428/tk2718Isup2.hkl

Additional supplementary materials:  crystallographic information; 3D view; checkCIF report

## Figures and Tables

**Table 1:** Hydrogen-bond geometry (Å, °)

*D*—H⋯*A*	*D*—H	H⋯*A*	*D*⋯*A*	*D*—H⋯*A*
C3*A*—H3*A*⋯O1*B*^i^	0.95	2.61	3.295 (8)	129
C13*B*—H13*B*⋯O3*B*^i^	1.00	2.69	3.541 (6)	143
C15*B*—H15*C*⋯O3*B*^i^	0.99	2.62	3.484 (6)	145
C8*A*—H8*A*1⋯O4*A*^ii^	0.99	2.68	3.452 (8)	135
C15*A*—H15*A*⋯O3*A*^i^	0.99	2.59	3.486 (6)	150
C3*B*—H3*B*⋯O1*A*^ii^	0.95	2.51	3.195 (8)	129
C8*B*—H8*B*1⋯O4*B*^ii^	0.99	2.56	3.394 (8)	143

Symmetry codes: (i) 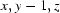; (ii) 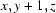.

**Fig. 1.**
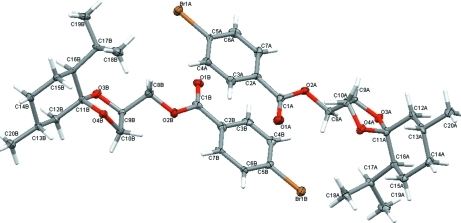
Displacement ellipsoid style view of the two molecules A and B of the title compound. Ellipsoid probability is at the 50% level.

**Fig. 2.**
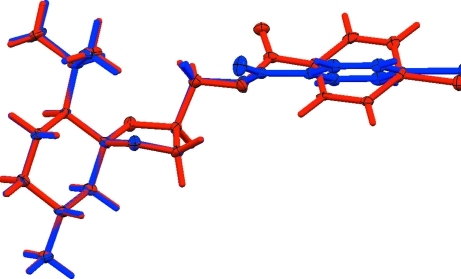
Overlay of the two crystallographically independent molecules.

**Fig. 3.**
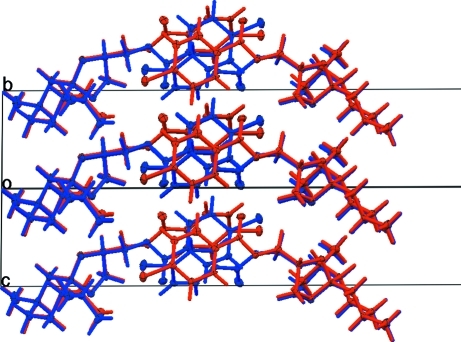
Packing view of the title compound, view down the (0 1 1) diagonal showing the pseudotranslation. Molecules A are shown in red, molecules B in blue.

**Fig. 4.**
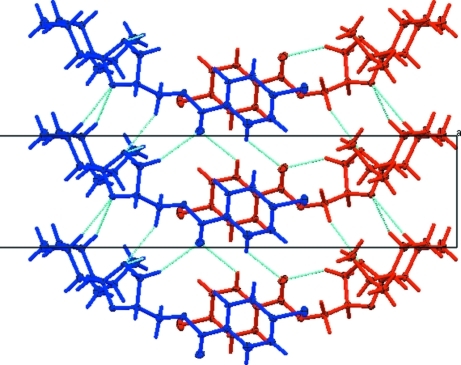
Packing view of the title compound with intermolecular C—H···O interactions shown (blue dashed lines). Molecules A are shown in red, molecules B in blue.

**Fig. 5.**
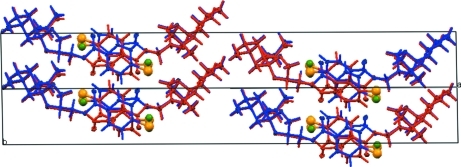
Q-peaks (yellow spheres) caused by correlation effects due to pseudo-translation and their positions with respect to the Br atoms (green smaller spheres). Q1, located close to Br1, is created by translation of Br2 and Q2 by translation of Br1 by half a unit cell along the (0 1 1) direction. View is down the direction of the pseudotranslation as in Fig. 3.

## supplementary crystallographic information

### Comment

The title structure was synthesized as part of a study of 3-carbon stereochemical moieties, specifically tri-substituted glycerol. Here menthone serves as a chiral auxiliary, freezing two carbons into a specific stereochemistry and influencing the stereochemistry of the third owing to the steric bulk of the menthone (Kiessling *et al.*, 2009*b*). Previously a different stereoisomer was isolated as the 3,5-dinitrobenzoate derivative and its crystal structure was published (Kiessling *et al.*, 2009*a*).

The starting material, glycerol menthonide, was originally prepared as an additive to spearmint gum by reaction of menthone with glycerol under acid catalysis (Greenberg, 1999). No further chemical analysis of the menthonide had been reported in the literature at that time. Later analysis revealed that glycerol menthonide exists in as many as six isomers, which proved to be difficult to separate (Kiessling *et al.* 2009*b*). However, conversion of the hydroxy group to an ester by reaction with 4-bromobenzoyl chloride yields a mixture of esters out of which the title compound crystallizes. Isolation of the crystals followed by sequential recrystallization from methanol/water yielded the title compound in > 97% purity in the form of colorless needles.

The title compound crystallizes with two crystallographically independent molecules in a monoclinic setting in the space group *C*2, Fig. 1. The two molecules, molecule A and B, are chemically identical and differ only in one on the torsion angles around the ester oxygen atom, C9—C8—O2—C1, which is -106.5 (7) ° in molecule A, and 146.1 (6) ° in molecule B. All other bonds, angles and torsion angles in both molecules are virtually identical, as can be seen in the overlay of the two molecules as shown in Fig. 2, and are within their expected ranges. The two molecules are not only very similar with respect to each other, they are also crystallographically related by a pseudotranslation found along the (0 1 1) diagonal of the unit cell (Fig. 3.). The glycerol menthonide of the two molecules are transformed into each other by a translation of half a unit cell along this direction. The *p*-bromo benzoate moieties, however, do not obey the pseudotranslation, thus causing a doubling of the unit cell with respect to a theoretical smaller primitive monoclinic cell with the dimensions a = 22.5949, b = 5.5763, c = 16.0718 and β = 108.193.

Packing in the structure of the title molecule is dominated by a combination weak interactions and van der Waals interactions. Via pairs of bifurcated C—H···O interactions between phenyl H atoms and the ester carbonyl O atoms molecules A and B form dimers (Fig. 4, Table 1). The dimers have local non-crystallographic inversion symmetry with the *p*-bromo benzoate moieties of the A and B molecules related by a pseudo inversion center in the middle of each dimer. The glycerol menthonide sections of the molecules are also interacting with each other with both oxygen atoms of the gylcerol units acting as acceptors for weak C—H···O interactions from aliphatic C—H and CH_2_ groups of neighboring glycerol menthonide moieties (Table 1). The connections are between like molecules and to both sides of the molecules, which leads to the formation of chains of molecules of either A or B that stretch parallel to the (0 1 0) direction. The combination of both types of C—H···O interactions leads to the formation of column shaped double chains as shown in Fig. 4. The outside of these columns is dominated by methyl, methylene and aromatic H atoms and the bromine atoms, and interactions between neighboring columns are limited to van der Waals interactions.

The refined Flack parameter of 0.000 (13) confirms the compound as a chiral and enantiopure molecule. The crystallographic assignment of the absolute stereochemistry is consistent with having started with (-)-menthone, and provides the stereochemistry of the acetal carbon and the esterified secondary alcohol of the glycerol chain. Specifically, the acetal carbon, C5, is *R* and the secondary alcohol, C2, is also *R*. This brings the bromobenzoate into approximately the same plane as the menthyl ring and *cis* to the isopropyl group.

### Experimental

All chemicals were purchased through ThermoFisher Inc. and used without further purification. Glycerol menthonide was prepared according to the published procedure (Greenberg 1999). GC/MS data was obtained using a Varian CP 3800 with Saturn 2000 ion trap MS. Column: Varian CP 5860, WCOT fused silica 30 m × 0.25 mm, coating CP-Sil. Carrier gas: He 1.2 ml/min. Temperature Program: initial temperature 473 K, ramp 20 K/min to 533 K hold 14.5 min. NMR data were obtained at Bucknell University using a Varian 600 MHz instrument and CDCl_3_, data are reported as p.p.m. from TMS and coupling constants are in Hz. Melting points were obtained on a MelTemp and are uncorrected. TLC was done with Analtech 2520 plates.

In a 50-ml round-bottom flask were placed glycerol menthonide (5.02 g, 22.0 mmol), 4-bromobenzoyl chloride (4.96 g, 23.0 mmol) and pyridine (10 ml). The flask was fitted with an air reflux condenser, drying tube and a magnetic stir bar. The flask was heated to reflux of the solvent while stirring for 2 h. The contents of the flask were then added to water (30 ml) and methyl *tert*-butyl ether (MTBE, 20 ml) and separated. The aqueous layer was extracted twice with MTBE (20 ml). The combined organic layers were washed with 10% HCl (2 × 15 ml), 10% Na_2_CO_3_ (2 × 15 ml) and saturated NaCl (15 ml), dried over MgSO_4_ and the solvent removed under vacuum to yield the crude product as an oil. To the oil was added methanol (10 ml) and the solution placed in a freezer for 72 hr. Vacuum filtration yielded the product as a white solid (1.19 g) which was 72% pure by GC/MS analysis. A portion of this solid was further purified by recrystallization from methanol/water to yield white needles, mp 353.5 – 354 K. TLC: *R*_f_ = 0.54 in 7% ethyl acetate/petroleum ether. GC: *R*_t_ = 12.11 min. IR: 2952, 1718, 1589, 1269, 1095, 1008, 849, 753. MS: 412 (18), 410 (18), 397 (34), 395 (35), 355 (48), 353 (48), 327 (100), 325 (86), 185 (36), 183 (32), 69 (45), expected for C_20_H_27_BrO_4_ 410.10. ^13^C NMR: 165.7, 131.7 (2), 131.3 (2), 128.7, 128.3, 113.1, 77.5, 65.9, 64.7, 48.3, 44.1, 33.5, 30.3, 24.1, 23.4, 23.2, 22.1, 18.1. ^1^H NMR: 7.92 (dt, J = 8.4, 1.8 Hz, 2H), 7.59 (dt, J = 9.0, 1.8 Hz, 2H), 4.53 (m, 1H), 4.48 (dd, J = 11.4, 4.2 Hz, 1H), 4.40 (dd, J = 11.4, 5.1 Hz, 1H), 4.09 (dd, J = 7.8, 6.9 Hz, 1H), 3.75 (dd, J = 7.8, 6.6 Hz, 1H), 2.24 (sept, J = 6.9 Hz, 1H)1.86 (ddd, J = 13.2, 2.4, 1.2 Hz, 1H), 1.71 - 1.77 (m, 1H), 1.56 - 1.68 (m, 2H), 1.34 - 1.46 (m, 2H), 1.01 (t, j = 12.9 Hz, 1H), 0.89 (d, J = 6.6 Hz, 3H), 0.87 (d, J = 7.2 Hz, 3H), 0.83 (d, J = 7.2 Hz, 3H), 0.86 - 0.90 (m, 1H).

### Refinement

Reflection 2 0 0 was obstructed by the beam stop and was omitted from the refinement. The structure shows pseudotranslation along the (0 1 1) diagonal. The *p*-bromo benzoate moieties do not obey the pseudotranslation and cause the doubling of the unit cell. The largest residual electron density peaks are located close to the bromine atoms, 0.84 Å from Br1 and 0.82 Å from Br2. The relatively large residual electron densities found (4.00 and 3.78 e Å^-3^) are associated with correlation effects due to the pseudotranslation exhibited by the structure. Q1, located close to Br1, is at a position that agrees with the position of Br2 translated along the direction of the pseudotranslation. Q2, on the other hand, reflects Br1 translated by half a unit cell along (0 1 1) (Fig. 5).

H atoms attached to carbon atoms were positioned geometrically and constrained to ride on their parent atoms, with C—H distances of 0.95 (CH_ar_), 0.99 (CH_2_), 0.98 (CH_3_) or 1.00 Å (C—H) and with *U*_iso_(H) = 1.2 *U*_eq_(C) or 1.5 *U*_eq_(C_methyl_) for methyl H.

### Crystal data

**Table:**

C_20_H_27_BrO_4_	*F*(000) = 1712
*M**_r_* = 411.33	*D*_x_ = 1.420 Mg m^−^^3^
Monoclinic, *C*2	Mo *K*α radiation, λ = 0.71073 Å
Hall symbol: C 2y	Cell parameters from 3693 reflections
*a* = 42.976 (7) Å	θ = 2.5–27.6°
*b* = 5.5763 (9) Å	µ = 2.16 mm^−^^1^
*c* = 16.072 (3) Å	*T* = 100 K
β = 92.618 (2)°	Needle, colourless
*V* = 3847.5 (11) Å^3^	0.50 × 0.05 × 0.03 mm
*Z* = 8	

### Data collection

**Table:**

Bruker SMART APEX CCD diffractometer	9230 independent reflections
Radiation source: fine-focus sealed tube	6765 reflections with *I* > 2σ(*I*)
graphite	*R*_int_ = 0.043
ω scans	θ_max_ = 28.3°, θ_min_ = 1.3°
Absorption correction: multi-scan (*SADABS*; Bruker, 2009)	*h* = −57→56
*T*_min_ = 0.588, *T*_max_ = 0.746	*k* = −7→7
17537 measured reflections	*l* = −21→21

### Refinement

**Table:**

Refinement on *F*^2^	Secondary atom site location: difference Fourier map
Least-squares matrix: full	Hydrogen site location: inferred from neighbouring sites
*R*[*F*^2^ > 2σ(*F*^2^)] = 0.058	H-atom parameters constrained
*wR*(*F*^2^) = 0.148	*w* = 1/[σ^2^(*F*_o_^2^) + (0.0759*P*)^2^] where *P* = (*F*_o_^2^ + 2*F*_c_^2^)/3
*S* = 1.02	(Δ/σ)_max_ = 0.001
9230 reflections	Δρ_max_ = 4.00 e Å^−^^3^
457 parameters	Δρ_min_ = −0.75 e Å^−^^3^
1 restraint	Absolute structure: Flack (1983), 3965 Friedel pairs
Primary atom site location: structure-invariant direct methods	Flack parameter: 0.000 (13)

### Special details

**Table:**

Geometry. All e.s.d.'s (except the e.s.d. in the dihedral angle between two l.s. planes) are estimated using the full covariance matrix. The cell e.s.d.'s are taken into account individually in the estimation of e.s.d.'s in distances, angles and torsion angles; correlations between e.s.d.'s in cell parameters are only used when they are defined by crystal symmetry. An approximate (isotropic) treatment of cell e.s.d.'s is used for estimating e.s.d.'s involving l.s. planes.
Refinement. Refinement of *F*^2^ against ALL reflections. The weighted *R*-factor *wR* and goodness of fit *S* are based on *F*^2^, conventional *R*-factors *R* are based on *F*, with *F* set to zero for negative *F*^2^. The threshold expression of *F*^2^ > σ(*F*^2^) is used only for calculating *R*-factors(gt) *etc*. and is not relevant to the choice of reflections for refinement. *R*-factors based on *F*^2^ are statistically about twice as large as those based on *F*, and *R*- factors based on ALL data will be even larger.

### Fractional atomic coordinates and isotropic or equivalent isotropic displacement parameters (Å^2^)

**Table:**

	*x*	*y*	*z*	*U*_iso_*/*U*_eq_	
Br1A	0.677987 (16)	0.69491 (10)	0.98525 (5)	0.02577 (19)	
Br1B	0.822411 (16)	0.57423 (10)	0.51465 (5)	0.02449 (18)	
C1A	0.79543 (14)	0.4707 (12)	0.7970 (4)	0.0172 (14)	
C2A	0.76758 (14)	0.5328 (11)	0.8445 (4)	0.0141 (12)	
C3A	0.74302 (14)	0.3702 (11)	0.8413 (4)	0.0165 (13)	
H3A	0.7448	0.2269	0.8099	0.020*	
C4A	0.71634 (15)	0.4125 (12)	0.8826 (4)	0.0167 (13)	
H4A	0.6998	0.2990	0.8814	0.020*	
C5A	0.71416 (17)	0.6295 (9)	0.9266 (5)	0.0119 (18)	
C6A	0.73825 (16)	0.7922 (11)	0.9304 (4)	0.0195 (14)	
H6A	0.7365	0.9360	0.9615	0.023*	
C7A	0.76521 (14)	0.7446 (12)	0.8881 (4)	0.0188 (14)	
H7A	0.7818	0.8571	0.8894	0.023*	
C8A	0.84603 (15)	0.6051 (12)	0.7615 (5)	0.0151 (15)	
H8A1	0.8517	0.7596	0.7359	0.018*	
H8A2	0.8419	0.4871	0.7163	0.018*	
C9A	0.87254 (10)	0.5184 (8)	0.8185 (3)	0.0130 (9)	
H9A	0.8746	0.6223	0.8691	0.016*	
C10A	0.86975 (11)	0.2539 (9)	0.8437 (3)	0.0169 (10)	
H10A	0.8786	0.2267	0.9009	0.020*	
H10B	0.8478	0.2005	0.8404	0.020*	
C11A	0.91262 (10)	0.2894 (9)	0.7673 (3)	0.0139 (9)	
C12A	0.93969 (12)	0.2590 (10)	0.8319 (3)	0.0172 (11)	
H12A	0.9557	0.3822	0.8221	0.021*	
H12B	0.9320	0.2852	0.8883	0.021*	
C13A	0.95462 (12)	0.0102 (10)	0.8282 (3)	0.0201 (11)	
H13A	0.9388	−0.1127	0.8423	0.024*	
C14A	0.96479 (11)	−0.0364 (10)	0.7392 (3)	0.0201 (11)	
H14A	0.9817	0.0764	0.7263	0.024*	
H14B	0.9731	−0.2014	0.7357	0.024*	
C15A	0.93810 (12)	−0.0064 (10)	0.6753 (3)	0.0172 (11)	
H15A	0.9220	−0.1289	0.6850	0.021*	
H15B	0.9458	−0.0324	0.6189	0.021*	
C16A	0.92351 (11)	0.2444 (9)	0.6798 (3)	0.0127 (10)	
H16A	0.9408	0.3608	0.6715	0.015*	
C17A	0.89885 (11)	0.2924 (9)	0.6090 (3)	0.0151 (9)	
H17A	0.8856	0.4287	0.6270	0.018*	
C18A	0.87701 (16)	0.0784 (15)	0.5892 (5)	0.0263 (15)	
H18A	0.8662	0.0337	0.6394	0.039*	
H18B	0.8893	−0.0582	0.5707	0.039*	
H18C	0.8616	0.1237	0.5450	0.039*	
C19A	0.91434 (13)	0.3708 (10)	0.5304 (3)	0.0234 (11)	
H19A	0.8983	0.4080	0.4869	0.035*	
H19B	0.9276	0.2411	0.5112	0.035*	
H19C	0.9271	0.5137	0.5422	0.035*	
C20A	0.98250 (12)	−0.0095 (12)	0.8913 (3)	0.0289 (13)	
H20A	0.9986	0.1052	0.8766	0.043*	
H20B	0.9910	−0.1725	0.8902	0.043*	
H20C	0.9757	0.0265	0.9473	0.043*	
C1B	0.70385 (15)	0.7839 (11)	0.6994 (4)	0.0161 (13)	
C2B	0.73214 (14)	0.7265 (13)	0.6536 (4)	0.0182 (13)	
C3B	0.75673 (15)	0.8902 (13)	0.6586 (4)	0.0199 (14)	
H3B	0.7550	1.0339	0.6898	0.024*	
C4B	0.78383 (15)	0.8412 (12)	0.6174 (4)	0.0174 (14)	
H4B	0.8008	0.9506	0.6204	0.021*	
C5B	0.78569 (19)	0.6330 (10)	0.5726 (5)	0.0149 (19)	
C6B	0.76197 (14)	0.4659 (12)	0.5682 (4)	0.0163 (13)	
H6B	0.7641	0.3207	0.5381	0.020*	
C7B	0.73489 (14)	0.5156 (11)	0.6091 (4)	0.0142 (13)	
H7B	0.7182	0.4042	0.6064	0.017*	
C8B	0.65383 (17)	0.6320 (10)	0.7341 (5)	0.0187 (18)	
H8B1	0.6496	0.8006	0.7493	0.022*	
H8B2	0.6560	0.5358	0.7858	0.022*	
C9B	0.62768 (10)	0.5353 (8)	0.6779 (3)	0.0136 (9)	
H9B	0.6255	0.6337	0.6259	0.016*	
C10B	0.63120 (11)	0.2705 (9)	0.6557 (3)	0.0163 (10)	
H10C	0.6230	0.2379	0.5982	0.020*	
H10D	0.6533	0.2198	0.6611	0.020*	
C11B	0.58757 (10)	0.3063 (9)	0.7291 (3)	0.0137 (9)	
C12B	0.56128 (12)	0.2708 (10)	0.6640 (3)	0.0168 (10)	
H12C	0.5693	0.2956	0.6079	0.020*	
H12D	0.5449	0.3919	0.6724	0.020*	
C13B	0.54689 (11)	0.0148 (10)	0.6691 (3)	0.0157 (11)	
H13B	0.5631	−0.1054	0.6551	0.019*	
C14B	0.53697 (11)	−0.0327 (9)	0.7582 (3)	0.0175 (10)	
H14C	0.5192	0.0726	0.7701	0.021*	
H14D	0.5299	−0.2010	0.7625	0.021*	
C15B	0.56317 (12)	0.0115 (10)	0.8228 (3)	0.0153 (11)	
H15C	0.5800	−0.1063	0.8149	0.018*	
H15D	0.5553	−0.0133	0.8791	0.018*	
C16B	0.57645 (11)	0.2651 (9)	0.8171 (3)	0.0124 (10)	
H16B	0.5584	0.3760	0.8231	0.015*	
C17B	0.60009 (12)	0.3292 (9)	0.8881 (3)	0.0180 (10)	
H17B	0.6117	0.4749	0.8707	0.022*	
C18B	0.6239 (2)	0.1343 (11)	0.9093 (5)	0.028 (2)	
H18D	0.6132	−0.0096	0.9282	0.042*	
H18E	0.6385	0.1913	0.9537	0.042*	
H18F	0.6354	0.0959	0.8598	0.042*	
C19B	0.58304 (13)	0.3940 (11)	0.9663 (3)	0.0257 (12)	
H19D	0.5983	0.4311	1.0117	0.039*	
H19E	0.5701	0.2583	0.9824	0.039*	
H19F	0.5698	0.5342	0.9550	0.039*	
C20B	0.51938 (12)	−0.0086 (11)	0.6060 (3)	0.0262 (12)	
H20D	0.5032	0.1069	0.6195	0.039*	
H20E	0.5109	−0.1716	0.6082	0.039*	
H20F	0.5264	0.0237	0.5500	0.039*	
O1A	0.79755 (11)	0.2908 (9)	0.7548 (3)	0.0252 (11)	
O2A	0.81834 (12)	0.6356 (6)	0.8087 (3)	0.0151 (13)	
O3A	0.90037 (7)	0.5265 (6)	0.7737 (2)	0.0152 (7)	
O4A	0.88748 (12)	0.1333 (6)	0.7838 (3)	0.0165 (13)	
O1B	0.70079 (11)	0.9560 (8)	0.7431 (3)	0.0222 (10)	
O2B	0.68186 (12)	0.6166 (8)	0.6883 (4)	0.0186 (13)	
O3B	0.59946 (7)	0.5436 (6)	0.7213 (2)	0.0158 (7)	
O4B	0.61311 (12)	0.1522 (7)	0.7151 (3)	0.0132 (12)	

### Atomic displacement parameters (Å^2^)

**Table:**

	*U*^11^	*U*^22^	*U*^33^	*U*^12^	*U*^13^	*U*^23^
Br1A	0.0183 (4)	0.0367 (4)	0.0226 (4)	0.0055 (4)	0.0042 (3)	−0.0042 (4)
Br1B	0.0170 (4)	0.0360 (4)	0.0207 (4)	0.0008 (4)	0.0030 (3)	−0.0012 (4)
C1A	0.013 (3)	0.023 (3)	0.015 (3)	0.002 (2)	0.000 (2)	0.004 (3)
C2A	0.022 (3)	0.009 (3)	0.012 (3)	0.003 (2)	−0.001 (2)	−0.002 (3)
C3A	0.021 (3)	0.008 (3)	0.020 (3)	−0.001 (2)	−0.001 (3)	−0.001 (2)
C4A	0.020 (3)	0.013 (3)	0.017 (3)	−0.005 (2)	−0.002 (3)	−0.002 (3)
C5A	0.009 (4)	0.017 (4)	0.009 (4)	0.0005 (19)	0.000 (3)	0.005 (2)
C6A	0.034 (4)	0.004 (3)	0.020 (3)	0.001 (3)	−0.002 (3)	0.000 (2)
C7A	0.015 (3)	0.015 (4)	0.026 (4)	−0.001 (3)	0.001 (3)	0.006 (3)
C8A	0.015 (3)	0.017 (3)	0.014 (3)	−0.001 (2)	0.005 (3)	0.002 (2)
C9A	0.015 (2)	0.014 (2)	0.010 (2)	−0.0014 (17)	0.0048 (17)	0.0005 (18)
C10A	0.017 (2)	0.017 (3)	0.016 (2)	0.0037 (18)	0.0025 (18)	0.0067 (19)
C11A	0.011 (2)	0.016 (2)	0.015 (2)	0.0002 (18)	−0.0002 (17)	0.0033 (19)
C12A	0.011 (2)	0.024 (3)	0.016 (2)	−0.002 (2)	−0.002 (2)	−0.002 (2)
C13A	0.015 (2)	0.031 (3)	0.014 (2)	−0.006 (2)	−0.008 (2)	0.007 (2)
C14A	0.014 (2)	0.029 (3)	0.018 (2)	0.009 (2)	0.0013 (19)	0.004 (2)
C15A	0.019 (3)	0.021 (3)	0.012 (2)	0.001 (2)	0.001 (2)	0.004 (2)
C16A	0.012 (2)	0.015 (3)	0.011 (2)	−0.002 (2)	0.0002 (19)	0.0048 (18)
C17A	0.018 (2)	0.015 (2)	0.012 (2)	−0.0008 (19)	−0.0034 (18)	0.0032 (19)
C18A	0.029 (4)	0.027 (3)	0.021 (3)	−0.010 (3)	−0.014 (3)	0.010 (3)
C19A	0.034 (3)	0.023 (3)	0.013 (2)	−0.003 (2)	0.002 (2)	0.006 (2)
C20A	0.021 (3)	0.052 (4)	0.013 (2)	0.005 (3)	−0.001 (2)	0.006 (2)
C1B	0.025 (3)	0.008 (3)	0.014 (3)	−0.005 (2)	−0.003 (3)	0.000 (2)
C2B	0.018 (3)	0.018 (4)	0.018 (3)	−0.001 (3)	−0.002 (3)	0.005 (3)
C3B	0.029 (4)	0.021 (3)	0.009 (3)	0.000 (3)	−0.002 (3)	0.004 (3)
C4B	0.018 (3)	0.020 (3)	0.014 (3)	0.001 (2)	−0.001 (3)	0.009 (3)
C5B	0.019 (4)	0.015 (4)	0.012 (4)	0.005 (2)	0.005 (4)	0.003 (2)
C6B	0.013 (3)	0.024 (3)	0.011 (3)	0.000 (2)	−0.003 (2)	−0.003 (3)
C7B	0.026 (3)	0.007 (3)	0.009 (3)	−0.004 (2)	−0.006 (2)	0.000 (2)
C8B	0.018 (4)	0.020 (4)	0.018 (4)	−0.001 (2)	0.003 (3)	−0.002 (2)
C9B	0.015 (2)	0.014 (3)	0.012 (2)	−0.0012 (17)	0.0026 (17)	−0.0013 (18)
C10B	0.017 (2)	0.017 (2)	0.015 (2)	−0.0012 (19)	0.0045 (18)	−0.0056 (19)
C11B	0.010 (2)	0.012 (2)	0.019 (2)	0.0002 (17)	0.0004 (17)	−0.0055 (19)
C12B	0.015 (2)	0.021 (3)	0.015 (2)	−0.002 (2)	−0.003 (2)	−0.001 (2)
C13B	0.011 (2)	0.020 (3)	0.017 (2)	0.001 (2)	−0.002 (2)	−0.002 (2)
C14B	0.016 (2)	0.018 (3)	0.018 (2)	−0.0035 (18)	0.0007 (19)	−0.003 (2)
C15B	0.015 (2)	0.017 (3)	0.014 (2)	0.000 (2)	0.005 (2)	−0.0024 (19)
C16B	0.011 (2)	0.015 (2)	0.011 (2)	0.003 (2)	0.0034 (18)	−0.0021 (18)
C17B	0.025 (3)	0.017 (2)	0.012 (2)	0.001 (2)	−0.0023 (19)	−0.0071 (19)
C18B	0.034 (4)	0.029 (4)	0.020 (4)	0.004 (2)	−0.007 (3)	−0.006 (2)
C19B	0.032 (3)	0.032 (3)	0.013 (2)	−0.001 (2)	0.001 (2)	−0.009 (2)
C20B	0.020 (3)	0.039 (3)	0.019 (3)	−0.005 (2)	−0.001 (2)	−0.009 (2)
O1A	0.026 (2)	0.022 (2)	0.028 (3)	−0.006 (2)	0.008 (2)	−0.011 (2)
O2A	0.017 (3)	0.012 (3)	0.016 (3)	0.0001 (14)	0.003 (2)	−0.0024 (14)
O3A	0.0153 (16)	0.0121 (18)	0.0187 (17)	0.0002 (12)	0.0058 (13)	0.0006 (14)
O4A	0.017 (3)	0.011 (3)	0.022 (3)	−0.0002 (14)	0.006 (2)	0.0029 (15)
O1B	0.025 (2)	0.020 (2)	0.022 (2)	−0.0031 (18)	0.0022 (19)	−0.008 (2)
O2B	0.013 (3)	0.018 (3)	0.025 (3)	−0.0051 (15)	0.004 (2)	−0.0064 (17)
O3B	0.0175 (16)	0.0105 (18)	0.0198 (17)	0.0008 (13)	0.0047 (13)	0.0000 (14)
O4B	0.016 (3)	0.008 (2)	0.016 (3)	0.0006 (14)	0.002 (2)	−0.0016 (15)

### Geometric parameters (Å, °)

**Table:**

Br1A—C5A	1.889 (7)	C20A—H20C	0.9800
Br1B—C5B	1.897 (8)	C1B—O1B	1.199 (8)
C1A—O1A	1.217 (8)	C1B—O2B	1.334 (7)
C1A—O2A	1.354 (8)	C1B—C2B	1.485 (9)
C1A—C2A	1.489 (8)	C2B—C7B	1.385 (10)
C2A—C7A	1.380 (10)	C2B—C3B	1.396 (9)
C2A—C3A	1.391 (9)	C3B—C4B	1.393 (9)
C3A—C4A	1.371 (9)	C3B—H3B	0.9500
C3A—H3A	0.9500	C4B—C5B	1.370 (9)
C4A—C5A	1.408 (9)	C4B—H4B	0.9500
C4A—H4A	0.9500	C5B—C6B	1.381 (9)
C5A—C6A	1.376 (9)	C6B—C7B	1.390 (8)
C6A—C7A	1.395 (9)	C6B—H6B	0.9500
C6A—H6A	0.9500	C7B—H7B	0.9500
C7A—H7A	0.9500	C8B—O2B	1.443 (9)
C8A—O2A	1.450 (8)	C8B—C9B	1.509 (8)
C8A—C9A	1.508 (8)	C8B—H8B1	0.9900
C8A—H8A1	0.9900	C8B—H8B2	0.9900
C8A—H8A2	0.9900	C9B—O3B	1.427 (5)
C9A—O3A	1.424 (5)	C9B—C10B	1.528 (6)
C9A—C10A	1.536 (6)	C9B—H9B	1.0000
C9A—H9A	1.0000	C10B—O4B	1.421 (6)
C10A—O4A	1.423 (7)	C10B—H10C	0.9900
C10A—H10A	0.9900	C10B—H10D	0.9900
C10A—H10B	0.9900	C11B—O4B	1.420 (6)
C11A—O4A	1.422 (6)	C11B—O3B	1.426 (6)
C11A—O3A	1.429 (6)	C11B—C12B	1.517 (7)
C11A—C16A	1.524 (6)	C11B—C16B	1.530 (6)
C11A—C12A	1.533 (7)	C12B—C13B	1.559 (8)
C12A—C13A	1.531 (8)	C12B—H12C	0.9900
C12A—H12A	0.9900	C12B—H12D	0.9900
C12A—H12B	0.9900	C13B—C20B	1.527 (7)
C13A—C20A	1.537 (7)	C13B—C14B	1.536 (7)
C13A—C14A	1.538 (7)	C13B—H13B	1.0000
C13A—H13A	1.0000	C14B—C15B	1.517 (7)
C14A—C15A	1.513 (7)	C14B—H14C	0.9900
C14A—H14A	0.9900	C14B—H14D	0.9900
C14A—H14B	0.9900	C15B—C16B	1.529 (8)
C15A—C16A	1.535 (7)	C15B—H15C	0.9900
C15A—H15A	0.9900	C15B—H15D	0.9900
C15A—H15B	0.9900	C16B—C17B	1.535 (7)
C16A—C17A	1.542 (7)	C16B—H16B	1.0000
C16A—H16A	1.0000	C17B—C18B	1.522 (9)
C17A—C19A	1.520 (6)	C17B—C19B	1.527 (7)
C17A—C18A	1.543 (9)	C17B—H17B	1.0000
C17A—H17A	1.0000	C18B—H18D	0.9800
C18A—H18A	0.9800	C18B—H18E	0.9800
C18A—H18B	0.9800	C18B—H18F	0.9800
C18A—H18C	0.9800	C19B—H19D	0.9800
C19A—H19A	0.9800	C19B—H19E	0.9800
C19A—H19B	0.9800	C19B—H19F	0.9800
C19A—H19C	0.9800	C20B—H20D	0.9800
C20A—H20A	0.9800	C20B—H20E	0.9800
C20A—H20B	0.9800	C20B—H20F	0.9800
			
O1A—C1A—O2A	124.4 (6)	C7B—C2B—C3B	120.2 (6)
O1A—C1A—C2A	124.0 (6)	C7B—C2B—C1B	122.1 (6)
O2A—C1A—C2A	111.6 (6)	C3B—C2B—C1B	117.7 (6)
C7A—C2A—C3A	120.2 (6)	C4B—C3B—C2B	119.5 (7)
C7A—C2A—C1A	122.6 (6)	C4B—C3B—H3B	120.3
C3A—C2A—C1A	117.1 (6)	C2B—C3B—H3B	120.3
C4A—C3A—C2A	121.3 (6)	C5B—C4B—C3B	119.1 (7)
C4A—C3A—H3A	119.3	C5B—C4B—H4B	120.5
C2A—C3A—H3A	119.3	C3B—C4B—H4B	120.5
C3A—C4A—C5A	117.9 (6)	C4B—C5B—C6B	122.6 (7)
C3A—C4A—H4A	121.0	C4B—C5B—Br1B	118.3 (6)
C5A—C4A—H4A	121.0	C6B—C5B—Br1B	119.1 (5)
C6A—C5A—C4A	121.4 (7)	C5B—C6B—C7B	118.2 (6)
C6A—C5A—Br1A	119.1 (5)	C5B—C6B—H6B	120.9
C4A—C5A—Br1A	119.5 (5)	C7B—C6B—H6B	120.9
C5A—C6A—C7A	119.6 (6)	C2B—C7B—C6B	120.5 (6)
C5A—C6A—H6A	120.2	C2B—C7B—H7B	119.8
C7A—C6A—H6A	120.2	C6B—C7B—H7B	119.8
C2A—C7A—C6A	119.5 (6)	O2B—C8B—C9B	106.8 (6)
C2A—C7A—H7A	120.3	O2B—C8B—H8B1	110.4
C6A—C7A—H7A	120.3	C9B—C8B—H8B1	110.4
O2A—C8A—C9A	109.6 (6)	O2B—C8B—H8B2	110.4
O2A—C8A—H8A1	109.8	C9B—C8B—H8B2	110.4
C9A—C8A—H8A1	109.8	H8B1—C8B—H8B2	108.6
O2A—C8A—H8A2	109.8	O3B—C9B—C8B	108.8 (4)
C9A—C8A—H8A2	109.8	O3B—C9B—C10B	103.9 (4)
H8A1—C8A—H8A2	108.2	C8B—C9B—C10B	113.9 (4)
O3A—C9A—C8A	108.2 (4)	O3B—C9B—H9B	110.0
O3A—C9A—C10A	104.0 (4)	C8B—C9B—H9B	110.0
C8A—C9A—C10A	113.7 (4)	C10B—C9B—H9B	110.0
O3A—C9A—H9A	110.3	O4B—C10B—C9B	103.2 (4)
C8A—C9A—H9A	110.3	O4B—C10B—H10C	111.1
C10A—C9A—H9A	110.3	C9B—C10B—H10C	111.1
O4A—C10A—C9A	103.0 (4)	O4B—C10B—H10D	111.1
O4A—C10A—H10A	111.2	C9B—C10B—H10D	111.1
C9A—C10A—H10A	111.2	H10C—C10B—H10D	109.1
O4A—C10A—H10B	111.2	O4B—C11B—O3B	105.4 (4)
C9A—C10A—H10B	111.2	O4B—C11B—C12B	111.7 (4)
H10A—C10A—H10B	109.1	O3B—C11B—C12B	108.6 (4)
O4A—C11A—O3A	105.5 (3)	O4B—C11B—C16B	109.4 (4)
O4A—C11A—C16A	109.9 (4)	O3B—C11B—C16B	110.4 (4)
O3A—C11A—C16A	110.4 (4)	C12B—C11B—C16B	111.3 (4)
O4A—C11A—C12A	111.5 (4)	C11B—C12B—C13B	111.6 (4)
O3A—C11A—C12A	108.9 (4)	C11B—C12B—H12C	109.3
C16A—C11A—C12A	110.6 (4)	C13B—C12B—H12C	109.3
C13A—C12A—C11A	112.4 (4)	C11B—C12B—H12D	109.3
C13A—C12A—H12A	109.1	C13B—C12B—H12D	109.3
C11A—C12A—H12A	109.1	H12C—C12B—H12D	108.0
C13A—C12A—H12B	109.1	C20B—C13B—C14B	111.4 (4)
C11A—C12A—H12B	109.1	C20B—C13B—C12B	109.9 (4)
H12A—C12A—H12B	107.9	C14B—C13B—C12B	109.5 (4)
C12A—C13A—C20A	110.8 (5)	C20B—C13B—H13B	108.7
C12A—C13A—C14A	109.0 (4)	C14B—C13B—H13B	108.7
C20A—C13A—C14A	110.8 (4)	C12B—C13B—H13B	108.7
C12A—C13A—H13A	108.7	C15B—C14B—C13B	112.4 (4)
C20A—C13A—H13A	108.7	C15B—C14B—H14C	109.1
C14A—C13A—H13A	108.7	C13B—C14B—H14C	109.1
C15A—C14A—C13A	112.0 (4)	C15B—C14B—H14D	109.1
C15A—C14A—H14A	109.2	C13B—C14B—H14D	109.1
C13A—C14A—H14A	109.2	H14C—C14B—H14D	107.9
C15A—C14A—H14B	109.2	C14B—C15B—C16B	112.1 (4)
C13A—C14A—H14B	109.2	C14B—C15B—H15C	109.2
H14A—C14A—H14B	107.9	C16B—C15B—H15C	109.2
C14A—C15A—C16A	111.5 (4)	C14B—C15B—H15D	109.2
C14A—C15A—H15A	109.3	C16B—C15B—H15D	109.2
C16A—C15A—H15A	109.3	H15C—C15B—H15D	107.9
C14A—C15A—H15B	109.3	C15B—C16B—C11B	109.2 (4)
C16A—C15A—H15B	109.3	C15B—C16B—C17B	114.0 (4)
H15A—C15A—H15B	108.0	C11B—C16B—C17B	115.3 (4)
C11A—C16A—C15A	109.7 (4)	C15B—C16B—H16B	105.8
C11A—C16A—C17A	115.0 (4)	C11B—C16B—H16B	105.8
C15A—C16A—C17A	113.1 (4)	C17B—C16B—H16B	105.8
C11A—C16A—H16A	106.1	C18B—C17B—C19B	109.1 (5)
C15A—C16A—H16A	106.1	C18B—C17B—C16B	114.6 (5)
C17A—C16A—H16A	106.1	C19B—C17B—C16B	110.0 (4)
C19A—C17A—C16A	110.6 (4)	C18B—C17B—H17B	107.6
C19A—C17A—C18A	109.7 (5)	C19B—C17B—H17B	107.6
C16A—C17A—C18A	114.1 (5)	C16B—C17B—H17B	107.6
C19A—C17A—H17A	107.4	C17B—C18B—H18D	109.5
C16A—C17A—H17A	107.4	C17B—C18B—H18E	109.5
C18A—C17A—H17A	107.4	H18D—C18B—H18E	109.5
C17A—C18A—H18A	109.5	C17B—C18B—H18F	109.5
C17A—C18A—H18B	109.5	H18D—C18B—H18F	109.5
H18A—C18A—H18B	109.5	H18E—C18B—H18F	109.5
C17A—C18A—H18C	109.5	C17B—C19B—H19D	109.5
H18A—C18A—H18C	109.5	C17B—C19B—H19E	109.5
H18B—C18A—H18C	109.5	H19D—C19B—H19E	109.5
C17A—C19A—H19A	109.5	C17B—C19B—H19F	109.5
C17A—C19A—H19B	109.5	H19D—C19B—H19F	109.5
H19A—C19A—H19B	109.5	H19E—C19B—H19F	109.5
C17A—C19A—H19C	109.5	C13B—C20B—H20D	109.5
H19A—C19A—H19C	109.5	C13B—C20B—H20E	109.5
H19B—C19A—H19C	109.5	H20D—C20B—H20E	109.5
C13A—C20A—H20A	109.5	C13B—C20B—H20F	109.5
C13A—C20A—H20B	109.5	H20D—C20B—H20F	109.5
H20A—C20A—H20B	109.5	H20E—C20B—H20F	109.5
C13A—C20A—H20C	109.5	C1A—O2A—C8A	117.1 (5)
H20A—C20A—H20C	109.5	C9A—O3A—C11A	109.1 (3)
H20B—C20A—H20C	109.5	C11A—O4A—C10A	105.8 (4)
O1B—C1B—O2B	122.8 (6)	C1B—O2B—C8B	119.5 (5)
O1B—C1B—C2B	125.2 (6)	C11B—O3B—C9B	109.2 (3)
O2B—C1B—C2B	111.9 (6)	C11B—O4B—C10B	106.0 (4)
			
O1A—C1A—C2A—C7A	−176.4 (7)	C1B—C2B—C7B—C6B	−178.8 (6)
O2A—C1A—C2A—C7A	5.4 (9)	C5B—C6B—C7B—C2B	−0.7 (10)
O1A—C1A—C2A—C3A	1.6 (10)	O2B—C8B—C9B—O3B	179.1 (4)
O2A—C1A—C2A—C3A	−176.6 (6)	O2B—C8B—C9B—C10B	63.7 (6)
C7A—C2A—C3A—C4A	−1.5 (10)	O3B—C9B—C10B—O4B	−22.7 (5)
C1A—C2A—C3A—C4A	−179.6 (6)	C8B—C9B—C10B—O4B	95.5 (5)
C2A—C3A—C4A—C5A	1.7 (10)	O4B—C11B—C12B—C13B	64.6 (5)
C3A—C4A—C5A—C6A	−1.7 (10)	O3B—C11B—C12B—C13B	−179.6 (4)
C3A—C4A—C5A—Br1A	−179.5 (5)	C16B—C11B—C12B—C13B	−57.9 (5)
C4A—C5A—C6A—C7A	1.5 (10)	C11B—C12B—C13B—C20B	176.8 (4)
Br1A—C5A—C6A—C7A	179.3 (5)	C11B—C12B—C13B—C14B	54.1 (6)
C3A—C2A—C7A—C6A	1.3 (10)	C20B—C13B—C14B—C15B	−174.8 (4)
C1A—C2A—C7A—C6A	179.2 (6)	C12B—C13B—C14B—C15B	−53.0 (6)
C5A—C6A—C7A—C2A	−1.2 (10)	C13B—C14B—C15B—C16B	55.9 (6)
O2A—C8A—C9A—O3A	−170.9 (4)	C14B—C15B—C16B—C11B	−56.9 (5)
O2A—C8A—C9A—C10A	74.2 (6)	C14B—C15B—C16B—C17B	172.5 (4)
O3A—C9A—C10A—O4A	−23.3 (5)	O4B—C11B—C16B—C15B	−65.8 (5)
C8A—C9A—C10A—O4A	94.1 (5)	O3B—C11B—C16B—C15B	178.7 (4)
O4A—C11A—C12A—C13A	65.0 (5)	C12B—C11B—C16B—C15B	58.0 (5)
O3A—C11A—C12A—C13A	−179.0 (4)	O4B—C11B—C16B—C17B	64.0 (5)
C16A—C11A—C12A—C13A	−57.5 (6)	O3B—C11B—C16B—C17B	−51.5 (5)
C11A—C12A—C13A—C20A	177.6 (4)	C12B—C11B—C16B—C17B	−172.1 (4)
C11A—C12A—C13A—C14A	55.4 (6)	C15B—C16B—C17B—C18B	44.4 (7)
C12A—C13A—C14A—C15A	−55.0 (6)	C11B—C16B—C17B—C18B	−83.1 (6)
C20A—C13A—C14A—C15A	−177.2 (5)	C15B—C16B—C17B—C19B	−79.0 (5)
C13A—C14A—C15A—C16A	57.1 (6)	C11B—C16B—C17B—C19B	153.6 (4)
O4A—C11A—C16A—C15A	−66.9 (5)	O1A—C1A—O2A—C8A	5.8 (10)
O3A—C11A—C16A—C15A	177.2 (4)	C2A—C1A—O2A—C8A	−176.0 (5)
C12A—C11A—C16A—C15A	56.5 (5)	C9A—C8A—O2A—C1A	−106.5 (6)
O4A—C11A—C16A—C17A	61.9 (5)	C8A—C9A—O3A—C11A	−118.0 (4)
O3A—C11A—C16A—C17A	−54.1 (5)	C10A—C9A—O3A—C11A	3.2 (5)
C12A—C11A—C16A—C17A	−174.7 (4)	O4A—C11A—O3A—C9A	18.3 (5)
C14A—C15A—C16A—C11A	−57.0 (5)	C16A—C11A—O3A—C9A	137.0 (4)
C14A—C15A—C16A—C17A	173.2 (4)	C12A—C11A—O3A—C9A	−101.4 (4)
C11A—C16A—C17A—C19A	150.3 (4)	O3A—C11A—O4A—C10A	−34.0 (5)
C15A—C16A—C17A—C19A	−82.6 (5)	C16A—C11A—O4A—C10A	−153.0 (4)
C11A—C16A—C17A—C18A	−85.5 (6)	C12A—C11A—O4A—C10A	84.2 (5)
C15A—C16A—C17A—C18A	41.6 (6)	C9A—C10A—O4A—C11A	35.1 (5)
O1B—C1B—C2B—C7B	175.2 (7)	O1B—C1B—O2B—C8B	−4.0 (10)
O2B—C1B—C2B—C7B	−3.2 (9)	C2B—C1B—O2B—C8B	174.5 (6)
O1B—C1B—C2B—C3B	−2.9 (10)	C9B—C8B—O2B—C1B	146.1 (6)
O2B—C1B—C2B—C3B	178.7 (6)	O4B—C11B—O3B—C9B	18.4 (5)
C7B—C2B—C3B—C4B	1.0 (10)	C12B—C11B—O3B—C9B	−101.4 (4)
C1B—C2B—C3B—C4B	179.1 (6)	C16B—C11B—O3B—C9B	136.4 (4)
C2B—C3B—C4B—C5B	0.2 (10)	C8B—C9B—O3B—C11B	−119.0 (4)
C3B—C4B—C5B—C6B	−1.7 (10)	C10B—C9B—O3B—C11B	2.8 (5)
C3B—C4B—C5B—Br1B	178.4 (5)	O3B—C11B—O4B—C10B	−33.6 (5)
C4B—C5B—C6B—C7B	1.9 (10)	C12B—C11B—O4B—C10B	84.1 (5)
Br1B—C5B—C6B—C7B	−178.1 (5)	C16B—C11B—O4B—C10B	−152.3 (4)
C3B—C2B—C7B—C6B	−0.7 (10)	C9B—C10B—O4B—C11B	34.6 (5)

### Hydrogen-bond geometry (Å, °)

**Table:**

*D*—H···*A*	*D*—H	H···*A*	*D*···*A*	*D*—H···*A*
C3A—H3A···O1B^i^	0.95	2.61	3.295 (8)	129.
C13B—H13B···O3B^i^	1.00	2.69	3.541 (6)	143.
C15B—H15C···O3B^i^	0.99	2.62	3.484 (6)	145.
C8A—H8A1···O4A^ii^	0.99	2.68	3.452 (8)	135.
C15A—H15A···O3A^i^	0.99	2.59	3.486 (6)	150.
C3B—H3B···O1A^ii^	0.95	2.51	3.195 (8)	129.
C8B—H8B1···O4B^ii^	0.99	2.56	3.394 (8)	143.

Symmetry codes: (i) *x*, *y*−1, *z*; (ii) *x*, *y*+1, *z*.
